# Gastroesophageal Reflux and Its Association With Atrial Fibrillation: A Traditional Review

**DOI:** 10.7759/cureus.10387

**Published:** 2020-09-11

**Authors:** Alaa Mohamed, Diego Ochoa Crespo, Gurleen Kaur, Ibtisam Ashraf, Mercedes Maria Peck, Ruchira Maram, Bilal Haider Malik

**Affiliations:** 1 Internal Medicine, California Institute of Behavioral Neurosciences & Psychology, Fairfield, USA; 2 Internal Medicine, Memorial Hermann Medical Center, Houston, USA; 3 Internal Medicine, Clinica San Martin, Azogues, ECU; 4 Neurology, California Institute of Behavioral Neurosciences & Psychology, Fairfield, USA; 5 Internal Medicine, Shalamar Institute of Health Sciences, Lahore, PAK; 6 Internal Medicine, Arogyasri Healthcare Trust, Hyderabad, IND

**Keywords:** a.fib, gerd, exercise, inflammation, hiatal hernia, ppis

## Abstract

Atrial fibrillation (AF) is a common arrhythmia, and gastroesophageal reflux disease (GERD) is a common gastroenterology disease; both are highly encountered daily in clinical practice. Since both share common predisposing factors, we can conclude that there is a link between them. To date, the precise mechanism of reflux disease as a possible cause of atrial fibrillation remains uncertain. However, some possibilities can be postulated, such as the inflammation process, and sympathovagal imbalance represents the main factors for how GERD can initiate AF. Vigorous aerobic exercise in healthy people can bring about acidic esophageal reflux, which is a common risk factor for AF. Various inflammatory markers such as C-reaction protein (CRP) and interleukins have been a central role in initiating AF. A large hiatal hernia (HH) can cause direct compression on the left atrium that is possibly predisposing to atrial arrhythmogenesis. It has been sporadically reported that using a proton pump inhibitor to treat GERD in patients with coexisting AF has a noticeable effect on decreasing symptoms of AF and recurrence with less cost and side effects.

## Introduction and background

Gastroesophageal reflux disease (GERD) is a digestive disease that is a common presenting complaint in the US, with a prevalence of 20% in the general population presenting in various ages in adults [1]. This prevalence of GERD within the US is continuing to increase and is now becoming one of the most chronic diseases which is commonly misdiagnosed and is often correlated with other diseases. GERD occurs when acidic stomach juices, fluids, and food are backed up from the stomach into the esophagus, causing extreme discomfort. Through the latest studies, it has been determined that GERD is considered a multifactorial disease that increases the provocation of various factors such as sliding hiatus hernia and unhealthy life-styles during a patient’s midlife age (ranging from 40 to 60) such as sleep apnea, overeating, obesity, smoking, and excessive alcohol intake [2,3,4,5].

GERD and its correlation with atrial fibrillation (AF) are being studied. AF is one of the most common cardiac arrhythmias that affects more than 2.2 million people in the US alone [6]. Studies have shown that there is an increase in the prevalence of AF in the older population and that there is quite possibly a link between AF and age [7]. There are several other factors that may be involved in the development of AF, such as sleep apnea, obesity, latent hypertension, gastroesophageal reflux disease, alcohol abuse, and systemic and local inflammation.

Since GERD and AF share some common features such as obesity, sleep apnea, and local inflammation, which increases with age, it is logical to conclude that there is a link between both diseases [8]. A 2012 study by Velagapudi et al. to determine the link between AF and acid reflux found that there is a possible link, although it is unclear. This study has shown that treating patients with acid-suppressive therapy has facilitated normal sinus rhythm in a subset of patients. It has been increasingly suggested that GERD, specifically the inflammation of the lower esophagus (esophagitis), induces the initiation and the perpetuation of AF [6].

This article aims to explain the relationship between AF and reflux disease and review relevant literature noting their association, and coherently produce information, relation, and association directed to healthcare workers.

## Review

It has become clear that GERD and AF are two illnesses that cross paths in various clinical practices, thus warranting further investigation. The overlap and link between the gastrointestinal symptoms and arrhythmias was first observed by Ludwig Roemheld and labeled “Roemheld Gastrocardiac Syndrome," also known as gastric-cardia, where esophagogastric acid was able to induce arrhythmia-related symptoms [8]. Finding the link and accurately diagnosing gastric-cardia is often tricky due to it being contributed to by various probable mechanisms that could cause this syndrome, such as inflammation within the close positioning of the esophagus and the atria, autoimmune disorders, common nerve innervations [6], vigorous aerobic exercise [9], mechanical influx, impediments in coronary blood flow, and hiatal hernias. 

Several studies have shown that proton pump inhibitors (PPIs), which are used as acid-suppressive therapy, can be possibly used as a viable substitute for the typical anti-arrhythmic medications used for the treatment of AF. It has been found that PPls can help induce normal sinus rhythm with the added benefit of it being lower in cost and with lower side-effects, although its full affectivity is yet to be determined.

Although the mechanism is yet unknown, there is some evidence from case reports through limited observational studies that report that reflux disease can cause and maintain paroxysmal AF (PAF). Acid reflux can cause irritations to the esophageal mucosa causing GERD, which leads to inflammation, which may play an essential role in the mechanism pathway that would result in PAF. The inflammation in the vagal nerves next to the esophagus influences AF, even in the absence of heart disease [10]. There is also increasing evidence indicating that PAF can appear in both young and middle-aged healthy athletes (e.g. cyclists, runners, and cross-country skiers). This evidence allows us to wonder if PAF appears in these athletes due to the link between AF and GERD. In this part of the article, we will try to explain in detail several physiological relationships between the upper gastrointestinal and cardiovascular systems.

Exercise, especially at a competitive level, is associated with AF

Through various studies, it has been demonstrated that aerobic exercise in healthy people can bring about esophageal acidic reflux that increases with time and the strength of the exercise. Several reflux mechanisms have been investigated, including decreased esophageal acid clearance, reduced gastric motility and absorption, lower-esophageal sphincter relaxation, reduced or disorganized peristaltic esophageal motility, and reduced GI blood flow [11]. A possible mechanism involved in exercise-induced gastroesophageal reflux (GER) is the structural integrity of the lower gastroesophageal junction (especially the flap valve) [12]. Collings et al. reported that within a study of 30 trained athletes, all of whom have gone through strenuous exercise, many had an increased stimulation of reflux or had symptoms associated with reflux. The study further indicated that as the athletes continued to eat light and healthy meals, their symptoms did not abate [7]. Continuing through the study, the degree of esophageal acidity was investigated in 10 healthy and fit sprinters who were asymptomatic. Following 80 minutes of moderate to hard intensity running, the athletes showed mild GERD signs. The pH was less than four in 4.9% of the fasting runners, and it was 17.2% in runners that had a light breakfast an hour before the run [13]. It is only logical to conclude from such a study that there is, in fact, a correlation between GERD and its symptoms and exercise. The lower esophageal inflammation (esophagitis) can lead to AF and other heart dysrhythmias. The two arguments together suggest that a regimen of excessive exercise may be conducive to AF mediated by acid reflux, an implicit hypothesis. It is highly reported that PPIs can be used in the treatment of exercise-induced GER [12,14,15].

Inflammation process and its effect on developing AF

**Figure 1:**
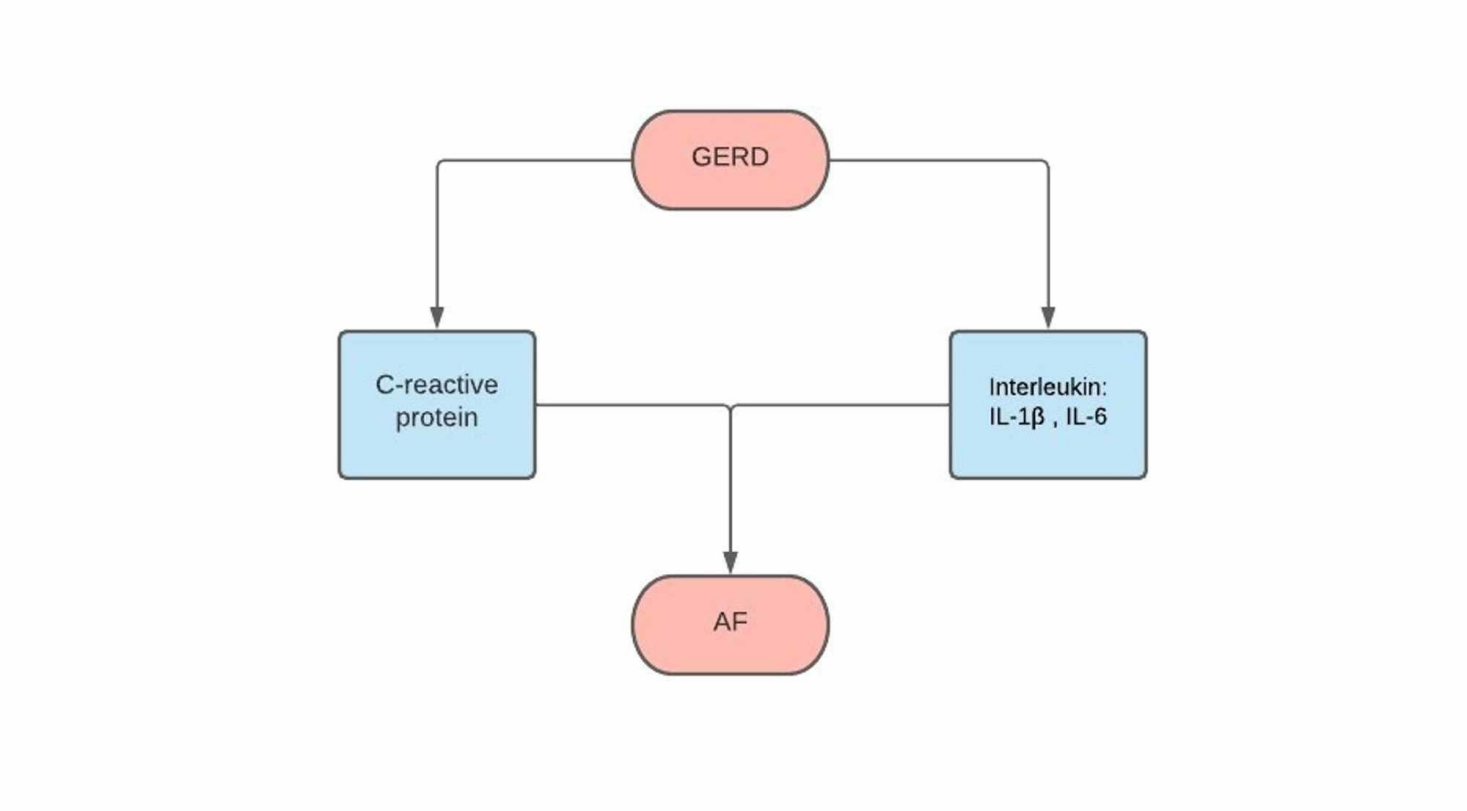
Inflammatory markers GERD: gastroesophageal reflux disease, AF: atrial fibrillation

Various studies have noted and studied the correlation between markers of inflammation and atrial fibrillation [6] - markers such as C-reaction protein (CRP) and interleukins (such as IL-1β and IL‐6) (Figure 1) [16]. GERD may also release a variety of inflammatory mediators, thus causing systemic inflammation. With the increase of the circulating CRP due to the inflammation, it may prompt the emergence of AF or symptoms of AF through the classic complement pathway activation that would lead to atrial tissue damage. This could also be caused by the CRP binding to phosphocholine, which would lead to alterations in sodium and calcium handling, which result in membrane dysfunction [17]. Another guide to the indication of chronic inflammation is an increase in CRP, which is in correlation with the occurrence [18,19], defibrillation efficacy [20], reappearance, and prognosis of AF [21,22].

Additionally, it has been noted through previous studies that acid reflux could cause inflammation through a separate mechanism that involves autonomic innervations within the esophageal mucosa. While the inflammation in the lower esophagus, the esophageal wall could be penetrated, which would, in turn, affect the vagal nerves. This could happen because of the proximity of the esophageal mucosa to the atria (in particular the left atrium) and how it could alter the local receptors [23]. This could consequently induce afferent-efferent reflux mechanisms of the cardiac rhythm, which could be due to the stimulation of the vagal nerves in a secondary fashion, thus encourage cardiac dysrhythmias [24].

Through the instances of GERD that could be caused by the inflammation in the lower esophageal, there are occasions where the inflammation penetrates the esophageal wall and cross into the thin layer of the pericardium, which causes atrial myocarditis and local pericarditis [25]. Through a study performed by Frustaci et al. septal biopsies were analyzed, and it was found that patients with lone atrial fibrillation had a presence of atrial lymphomononuclear infiltrates [26].

Autonomic influence induces AF

In certain instances, it has been found that an episode of atrial arrhythmia can sometimes be induced by any mechanical influence in the throat such as swallowing and even food passing through the esophagus into the stomach [27,28,29]. Paroxysmal AF, in turn, can also be stimulated by various gastrointestinal mechanisms, including defecation, bloating of the abdomen, and the mechanism involving eating and swallowing.

Cardiac responses associated with the nerves of the esophagus have been reported as early as the 1990s through a study performed by Tougas et al. [30], who was able to investigate the correlation of the effect of the mechanical stimulation of the esophagus and the heart rate and its variables. This was done using an analysis called the power spectrum of frequency domain analysis. Through this form of analysis, they found an increase in high-frequency component (HF) and a decrease in low-frequency component (LF) associated with a basic heart rate, which is caused by the mechanical esophageal stimulation.

Though the study mentioned above, it has been ascertained that when the autonomic nervous pathways are imbalanced (sympathovagal imbalance), it causes AF while being associated with GERD. Both parasympathetic and sympathetic pathways have a central role in AF, but parasympathetic pathways play a more significant role [31].

Hiatal hernia and mechanical influence

In the event that a patient has a hiatal hernia (HH), the food and acid rise, consequently rising the chest cavity pressure, in addition to the protrusion itself which can press upon the left atrium. It is found that HH is most prevalent (70%) in age groups older than 70 years of age, (although, the association between AF and HH may be coincidental as this age group is already prone to AF) while only 10% of patients are younger than 40 years of age. Having an HH can worsen or induce symptoms of GERD regardless of age [32].

Patients with HH can be treated surgically with fundoplication either laparoscopic Nissen or Belsey Mark IV fundoplication, and it has been observed that patients who complete the surgery also have a reduction in the instances or symptoms of AF [33,34]. This could lead to the logical conclusion that there is a correlation between the HH and AF, although it has not been thoroughly studied and investigated.

**Figure 2:**
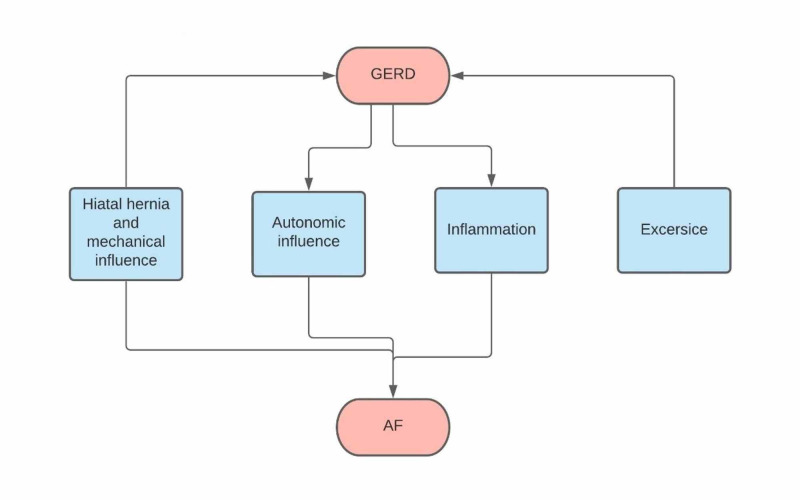
flow chart GERD: gastroesophageal reflux disease, AF: atrial fibrillation

Although inconclusive, there are two possible mechanisms that can be used to justify why the treatment of the HH and GERD can relieve the associated AF. The first speculated mechanism is the presence of an exceedingly giant HH that can affect the left atrium of the heart by direct pressure, resulting in decreased blood supply to the heart, causing relative ischemia which may lead to reentry and arrhythmia [35]. The second possible mechanism might be due to inflammation of the lower esophagus caused by the HH acid reflux, thus leading to AF (Figure 2).

PPIs and their effect on AF

As discussed previously, it has been determined that the best course of treatment for GERD is through protein pump inhibitors (PPIs), which in turn invariably resulted in the reduction of AF and its symptoms and can even completely stop the emergence of paroxysmal AF events [36,37]. This correlation has been determined to center around the effect of acid reflux and how it responds to PPI and its effect on paroxysmal AF.

The mechanism in which PPI functions goes beyond the simple suppression of stomach acid. It works by inhibiting the proton pump (K+H+ ATPase) located in the gastric mucosa [38]. It is also acid inhibition by its anti-inflammatory and antioxidant effects. The PPI does not only induce anti-inflammatory responses but also causes the suppression of the production of hydroxyl radical (-OH) on a cellular level (in vitro) [39,40]. Furthermore, there have been studies that concluded that the PPI treatment also has anti‐inflammatory actions acts against endothelial cells, epithelial cells, and the leukocytes, which are mediated in part by intracellular pH homeostasis and by modulating tissue [38]. 

It has also been found that there are functionally similar proteins of the gastric K+H+ATPase within the heart on protein and transcript levels [41]. After being compared, it has been found that the specific binding sites of the PPI are similar in both types of tissues. Thus it is found that the PPI acts as an anti-arrhythmic and cardioprotective agent that functions within the body as more than a simple antacid treatment, especially for the portion of the patients with symptoms of AF, which could be triggered by symptoms of GERD [42].

In a more uncommonly studied pathway, it has been noticed that GERD may elicit an autoimmune response through the pathogenesis of AF. Autoantibodies attack the myosin heavy chain in patients with lone AF [43].

Another less commonly studied pathway has been hypothesized that the coronary blood flow may decrease in patients with GERD and ischemic heart disease [44]. It could be logically concluded through this observation that chronic atrial ischemia could be, in turn, a trigger for AF [45].

## Conclusions

Gastroesophageal reflux disease and atrial fibrillation are two diseases that are encountered within the clinical practice at a high rate where it is easy to recognize that there could be a logical correlation between them. Both diseases are profoundly affected by many life-style related diseases, which are characterized commonly by persistent inflammation and sympathetic and parasympathetic imbalance. Through the treatment of GERD using PPIs, it has been ascertained that it also has a noticeable effect on AF and its symptoms which could be logically concluded that PPIs could be used as a more cost-effective method in treating AF.

Based on the evidence so far presented through the discussion, it has been strongly suggested that acid reflux and other GERD symptoms are a risk factor for AF. Although there are not enough studies to support this claim, there is enough data to support the correlation of the esophageal inflammation and AF. It remains unclear the extent to which the esophageal inflammation caused by acid exposure has a gradual effect on the sympathetic and parasympathetic system and the type of AF it induces. A few case reports and observational studies have reported a relation but an actual mechanism between GERD and AF is yet to be established, and more studies are required to explore the exact mechanism in which this link occurs.
